# Food reward sensitivity, impulsivity, and weight change during and after a 3-month weight loss program

**DOI:** 10.1371/journal.pone.0243530

**Published:** 2020-12-11

**Authors:** Kathryn M. Ross, Abraham Eastman, Umelo A. Ugwoaba, Kathryn E. Demos, Jason Lillis, Rena R. Wing

**Affiliations:** 1 Department of Clinical & Health Psychology, College of Public Health and Health Professions, University of Florida, Gainesville, Florida; 2 Department of Psychiatry and Human Behavior, Warren Alpert Medical School of Brown University, The Miriam Hospital/Weight Control and Diabetes Research Center, Providence, Rhode Island; University of Tennessee Health Science Center, UNITED STATES

## Abstract

**Background:**

Greater sensitivity to food rewards and higher levels of impulsivity (and an interaction between these variables, termed “reinforcement pathology”) have been associated with obesity in cross-sectional studies. Less is known regarding how these constructs may impact attempts at weight loss or longer-term weight loss maintenance.

**Methods:**

We provided 75 adults (69%Female, 84%White, age = 50.8y, BMI = 31.2kg/m^2^) with a 3-month Internet-based weight loss program and assessed weight, food reward sensitivity (via the Power of Food Scale [PFS]), and impulsivity (via Go No-Go [GNG] and Delay Discounting [DD] computer tasks) at baseline and at Months 3, 6, 9, and 12. No additional intervention was provided Months 3–12. Multi-level mixed-effect models were used to examine changes in PFS, GNG, and DD over time and associations between these measures and weight loss/regain.

**Results:**

Participants lost 6.0±1.1kg Months 0–3 and regained 2.4±1.1kg Months 3–12. Across time points, higher PFS scores were associated with higher weight, *p* = .007; however, there were no significant associations between GNG or DD and weight nor between the interactions of PFS and GNG or DD and weight, *p*s>.05. There were significant decreases from Months 0–3 in PFS, GNG, and DD, *p*s < .05; however, neither baseline values nor changes were significantly associated with weight change and there were no significant associations between the interactions of PFS and GNG or DD and weight change, *p*s>.05.

**Conclusion:**

Results demonstrated an association between food reward sensitivity and weight. Further, decreases in both food reward sensitivity and impulsivity were observed during an initial weight loss program, but neither baseline levels nor improvements were associated with weight change. Taken together, results suggest that the constructs of food reward sensitivity, impulsivity, and reinforcement pathology may have limited clinical utility within behavioral weight management interventions. Future intervention studies should examine whether food-related impulsivity tasks lead to a similar pattern of results.

## Introduction

Researchers have argued that the rising prevalence of overweight and obesity in the United States has been driven by an “obesogenic” food environment, including widespread availability of low-cost, highly-palatable, and high-calorie foods [1]. This food environment may be especially problematic for individuals who find food highly rewarding or motivating. Indeed, higher sensitivity to food rewards has been found to be associated with greater caloric intake [2] and obesity in several cross-sectional studies [2–4]. Moreover, one longitudinal study has demonstrated that higher food reward sensitivity predicted greater weight gain over one year in adults without obesity [5].

Differences in impulsivity may also impact how individuals interact with an obesogenic food environment. In this literature, impulsivity has been used to describe both a preference for immediate versus delayed rewards (e.g., discounting the value of future rewards, or “delay discounting”) and challenges with inhibitory control (e.g., inability to suppress the urge to act, or “behavioral impulsivity”). Higher levels of behavioral impulsivity have been associated with greater food intake during a lab-based eating study [6] and, in several cross-sectional studies, higher levels of both behavioral impulsivity and delay discounting have been shown to be associated with obesity [7–10].

Several researchers have also argued that there may be an important interaction to consider between food reward sensitivity and impulsivity (termed “reinforcement pathology”), such that higher levels of impulsivity strengthen the association between food reward sensitivity and eating intake/obesity [11–13]. Specifically, individuals low in impulsivity may be able to override greater food reward sensitivity when attempting to regulate weight, while individuals with high levels of both impulsivity and food reward sensitivity may have the greatest challenges with regulating eating intake and weight in obesogenic food environments, putting them at high risk for excess weight gain.

Two lab-based eating studies have provided support for this hypothesis: both found that delay discounting moderated the association between food reward sensitivity and palatable food intake, such that the association between food reward sensitivity and food intake was stronger in individuals with greater delay discounting [14,15]. In both of these studies, individuals who were high in both food reward sensitivity and delay discounting showed the highest intake of palatable foods, and individuals who were low in delay discounting showed little association between food reward sensitivity and intake. One other lab-based eating study found no significant associations between food reward sensitivity and impulsivity (or their interaction) and palatable food intake following consumption of an oatmeal pre-load [16]; however, this study reported a post-hoc association between the interaction of food reward sensitivity and impulsivity and both total food consumed and the amount of an oatmeal pre-load consumed. Beyond eating intake, the interaction between food reward sensitivity and delay discounting has been demonstrated to be associated with BMI in a cross-sectional study [17], and a longitudinal study demonstrated that the interaction between implicit snack food preference and behavioral impulsivity predicted weight gain over one year in female undergraduate college students without obesity [18].

Less is known regarding how food reward sensitivity, impulsivity, and their interaction impact weight loss outcomes. Three studies have investigated the association between food reward sensitivity and weight loss in adults taking part in a behavioral weight management program, but none of these studies included measures of impulsivity and thus they could not investigate the interaction between food reward sensitivity and impulsivity. Two of the three studies, conducted by Moore and colleagues [19] and O’Neil and colleagues [20], demonstrated no association between baseline food reward sensitivity and weight loss. The third, conducted by Theim and colleagues [21], found that higher sensitivity to food rewards at baseline was associated with *greater* weight loss. Participants in all three of these studies demonstrated decreases in food reward sensitivity during the behavioral weight loss program and, in the studies by O’Neil and colleagues and Theim and colleagues, larger decreases in food reward sensitivity were associated with greater weight losses (the study by Moore and colleagues did not test this association). In contrast, a study in adults undergoing bariatric surgery demonstrated significant reductions in food reward sensitivity at a postoperative assessment (conducted an average of 16 months after surgery), but these decreases were not significantly associated with magnitude of weight loss [22].

Three additional studies investigated associations between impulsivity and weight loss; however, none of these studies assessed food reward sensitivity and thus they could not investigate an interaction between impulsivity and food reward sensitivity. The first, conducted in 26 children attending an 8-week behavioral weight management program, found that behavioral impulsivity was positively associated with weight at baseline and that higher behavioral impulsivity at baseline predicted less weight loss during the intervention [23]. A second study, conducted with 47 adolescent boys completing an 8-week weight loss camp, measured both behavioral impulsivity and delay discounting [24]. Replicating the first camp study, this second study found that higher behavioral impulsivity at baseline was associated with less weight loss. No significant associations were observed, however, between baseline delay discounting and weight loss outcomes. Further, results demonstrated significant decreases in behavioral impulsivity and delay discounting from baseline to immediate posttest, but only decreases in behavioral impulsivity were associated with greater weight loss. A third study conducted in adults undergoing bariatric surgery found that greater decreases in behavioral impulsivity (but not in delay discounting) between a pre-surgical appointment and a 6-month postsurgical follow-up appointment were associated with greater weight losses during the same time period [25].

Taken together, the existing literature shows a clear association between food reward sensitivity and impulsivity and obesity in cross-sectional studies, and that food reward sensitivity, impulsivity, and their interaction are associated with palatable food intake in laboratory settings. Studies investigating the independent roles of food reward sensitivity and impulsivity in weight loss have shown mixed results and, to date, no studies have examined the interaction between food reward sensitivity and impulsivity in the context of weight loss. Thus, the current study proposed to investigate associations between food reward sensitivity, impulsivity, and their interaction and weight change during and after a 3-month, Internet-based behavioral weight loss program. We hypothesized that food reward sensitivity would be positively associated with body weight across time points (replicating previous cross-sectional studies), and that higher food reward sensitivity at baseline would be associated with less weight loss and greater weight regain. We also hypothesized that participants would experience a decrease in food reward sensitivity during the 3-month intervention, and that this decrease would be associated with greater weight loss during intervention and less weight regain during a 9-month follow-up period. We hypothesized that the same pattern of results would be observed for impulsivity (assessed both as behavioral impulsivity and delay discounting). Finally, we hypothesized that there would be a significant interaction between food reward sensitivity and impulsivity (again assessed as both behavioral impulsivity and delay discounting), and that we would observe a similar pattern of effects between this interaction and both weight across time points and weight change over time.

## Methods

The current study conducted analyses on data collected during the implementation of a 12-week, Internet-based weight loss program within a workplace healthcare rewards program [26]. Study procedures were approved by The Miriam Hospital Institutional Review Board, and written informed consent was obtained from all study participants. Study participants were provided with a 12-week, Internet-based behavioral weight loss program followed by a 9-month observational “maintenance” period during which no additional intervention was provided. In-person assessments occurred at Baseline (Month 0), Month 3 (immediate posttest), Month 6, Month 9, and Month 12.

### Participants

Full details regarding participant recruitment, inclusion/exclusion criteria, and baseline participant characteristics have been published previously [26]. In brief, participants were 75 adults (age: 18–70 years old, mean ± SD age = 50.76 ± 10.38 years), with overweight or obesity (body mass index [BMI] ≥ 25 kg/m^2^, mean ± SD baseline BMI = 31.19 ± 4.41 kg/m^2^) who were employees or dependents of employees of a large healthcare corporation in Providence, RI and who reported having access to a computer connected to the internet at home. Participants were excluded from the initial intervention implementation study if they reported being pregnant, postpartum, or that they planned to become pregnant during the study period or if they had any medical comorbidities that contraindicated weight loss. There was not a maximum BMI used for exclusion; however, participants with weights over 180 kg were excluded due to a restriction of the in-home scales provided as part of the intervention. Overall, 69.3% of the sample reported identifying as female and, in terms of race/ethnicity, 84.0% reported identifying as non-Hispanic White, 5.3% as non-Hispanic African American or Black, 2.7% as Hispanic, and 8.0% selected other or multiple categories.

### Initial weight loss program

As part of the Internet-based weight loss program, participants were provided with 12 weekly multimedia weight loss lessons that presented standard behavioral weight loss strategies adapted from the Diabetes Prevention Program [27] and Look AHEAD [28]. Participants were encouraged to reduce caloric intake (with initial daily calorie goals ranging from 1,200–1,800 kcal/day, based on baseline weight) and to gradually increase engagement in moderate-intensity physical activity (to eventually reach a goal of 200 minutes/week). Participants were provided with a calorie reference book, paper self-monitoring records, and an in-home scale. Participants were asked to self-monitor caloric intake, physical activity, and weight daily, and to self-report this data on the study website at the end of each week. At the start of each new week, an automated algorithm used this self-report data to generate targeted feedback messages to participants based on their self-monitoring habits and goal achievement.

### Maintenance period

After the end of the 12-week program, participants were encouraged to continue to self-monitor their caloric intake, physical activity, and weight daily. Participants were also asked to continue to report summaries of their self-monitoring habits via the study website weekly, but no additional intervention was provided (no tailored feedback was provided and participants no longer had access to the intervention content previously posted on the study website, including the weekly lessons).

### Compensation

Small financial incentives were provided to support submission of weekly data via the study website. Specifically, participants were eligible to receive between $1 and $10 each week (incentives varied on a schedule unknown to participants) with an average of $3.50 per week over the study year and a maximum total of $156 [29]. These incentives were collected in an online “bank,” and provided to participants at the in-person assessment visits. Moreover, participants received $20 for completing each in-person assessment visit. Finally, as an existing part of the healthcare rewards program, all employees and dependents of the healthcare organization were eligible to receive $250 for completing their annual physical at their physician’s office and having either a) a BMI ≤ 30 kg/m2 or b) lost either ≥5% of their starting weight or ≥17 lbs; the Internet-based weight management intervention was one of the possible programs that individuals could use to lose weight toward this goal [26].

### Measures

#### Height and weight

Participants were asked to remove shoes, empty their pockets, and to remove any bulky clothing (e.g., sweaters or coats) prior to measurement of height and weight. Height was measured at baseline, to the nearest 0.1 cm, by a trained study staff member using a wall-mounted stadiometer. Weight was assessed at all assessment visits by trained study staff members to the nearest 0.1 kg, using a calibrated Tanita BWB-800 digital scale (Arlington Heights, Illinois, USA).

#### Food reward sensitivity

Food reward sensitivity was measured at all assessment time points using the Power of Food Scale (PFS), a 15-item self-report questionnaire [30,31] that has been used in several previous studies investigating food reward sensitivity in relation to eating intake and obesity [15,19,20,22]. This measure assesses sensitivity to food rewards by having participants respond to statements using 5-point Likert scales (ranging from 1 = “I don’t agree” to 5 = “I strongly agree”), across three subscales: “Food Available” (e.g., “I get more pleasure from eating than I do from almost anything else”), “Food Present” (e.g., “If I see or smell a food I like, I get a powerful urge to have some”), and “Food Tasted” (e.g. “When I eat delicious food I focus a lot on how good it tastes”) [31]. Participants were asked to complete a printed version of this questionnaire, along with other self-report questionnaires used in the parent study, while attending each in-person assessment visit. For scoring, items on each subscale were averaged across the subscale, and a total score was calculated using an overall average (through calculating a sum of all items and dividing by 15).

#### Impulsivity

Impulsivity was assessed in two ways (with both methods using computerized tasks completed on study laptops during each in-person assessment visit). First, behavioral impulsivity was assessed at all assessment time points via a computerized Go/No-Go task programmed using E-Prime 2.0 Professional software (Psychology Software Tools, Inc., Sharpsburg, PA, USA). The design of this task was based on a task used by Kielhl and colleagues [32] and was similar to tasks used in other studies investigating associations between behavioral impulsivity and obesity [18,23–25]. Using a study computer, participants were instructed to press a button each time a “Go” stimulus appeared on the computer screen (in order to establish a pre-potent response), and to not press the button every time that a “No-Go” stimulus appeared on the screen (in order to establish ability to inhibit response). The letter “X” was used as the No-Go stimulus; this stimulus remained constant and was not dependent upon other stimuli [33]. The “Go” stimuli were randomly-selected letters other than “X”. A 1 Hz serial stream of letters were presented for 100 ms each, with an inter-stimulus interval of 900 ms. A total of 512 letters were presented, consisting of 80% “Go” stimuli and 20% “No-Go” stimuli. Behavioral impulsivity was operationalized as the number of errors of commission made on the “No-Go” condition (that is, the number of times that participants incorrectly hit the button when “X” appeared).

Second, delay discounting was assessed at all time points via a computer task developed by Koffarnus and Bickel [34], similar to methods used in previous studies investigating delay discounting in relation to eating intake and obesity [15,16,24,25]. In this task, participants chose between hypothetically receiving a larger sum ($100 or $1,000) after a fixed time delay (1 day, 1 week, 1 month, 6 months, and 1 year) or a smaller but immediate sum of money that varied across trials. An adjusting algorithm determined the smaller amount of immediately available money for each trial. For each time delay, participants first had to choose between receiving the delayed larger sum (e.g. $1,000) or half of that amount (e.g. $500) available immediately. If the participant selected the delayed choice, the amount of the immediate reward then adjusted up by half (e.g., to $750). If the participant selected the immediate choice, the amount of the immediate reward then adjusted down by half (e.g., to $250). This process continued for five trials per time delay, with the immediate amount adjusting by half the amount of the previous adjustment. As an example, if a participant had selected the $1000 with delay over the immediate $750, the subsequent choice would be between $1000 after the specified delay and $875 immediately. Indifference points were defined as the points at which larger delayed rewards were equivalent to smaller immediate rewards. This resulted in 32 potential indifference points evenly spaced between $0 and the larger sum. Area under the curve (AUC) methods were used to assess discounting rate for the $100 and $1000 conditions separately [35], with lower AUCs representing greater discounting.

### Statistical analyses

All analyses were conducted using SAS version 9.4 [36]. Multi-level mixed-effects models (SAS proc MIXED) were used to examine changes in food reward sensitivity, behavioral impulsivity, delay discounting, and weight over time, and to investigate main effects of food reward sensitivity and impulsivity (and their interaction, representing the construct of reinforcement pathology) on weight across assessments (i.e., pooled across all assessment points, replicating previous cross-sectional analyses) and over time (i.e., changes in weight between assessment visits). Throughout these models, a repeated-measures framework was used, with “participant” modeled as a random effect (allowing for variation within-person, over time) [37]. All other model parameters were modeled as fixed effects (model fit, using the Akaike information criterion, was assessed with these parameters entered as random effects; however, there was no evidence of improved model fit for any models including additional random effects and thus results of the models using fixed effects are presented). Given the known non-linear trajectory of weight change during and after weight management programs, wherein most individuals lose weight during the initial program but begin to regain weight after the end of the program [38,39], associations between food reward sensitivity, impulsivity, and weight change were examined separately during the initial weight loss program and the maintenance period. All available data were used in the analyses, with maximum likelihood estimation used to handle missing data (a restricted maximum likelihood approach was used to allow for unbiased estimates). As a post-hoc analysis, correlations were used to examine associations between baseline PFS scores, No-Go errors on the Go/No-Go task, and delay discounting scores.

## Results

**Fig 1:**
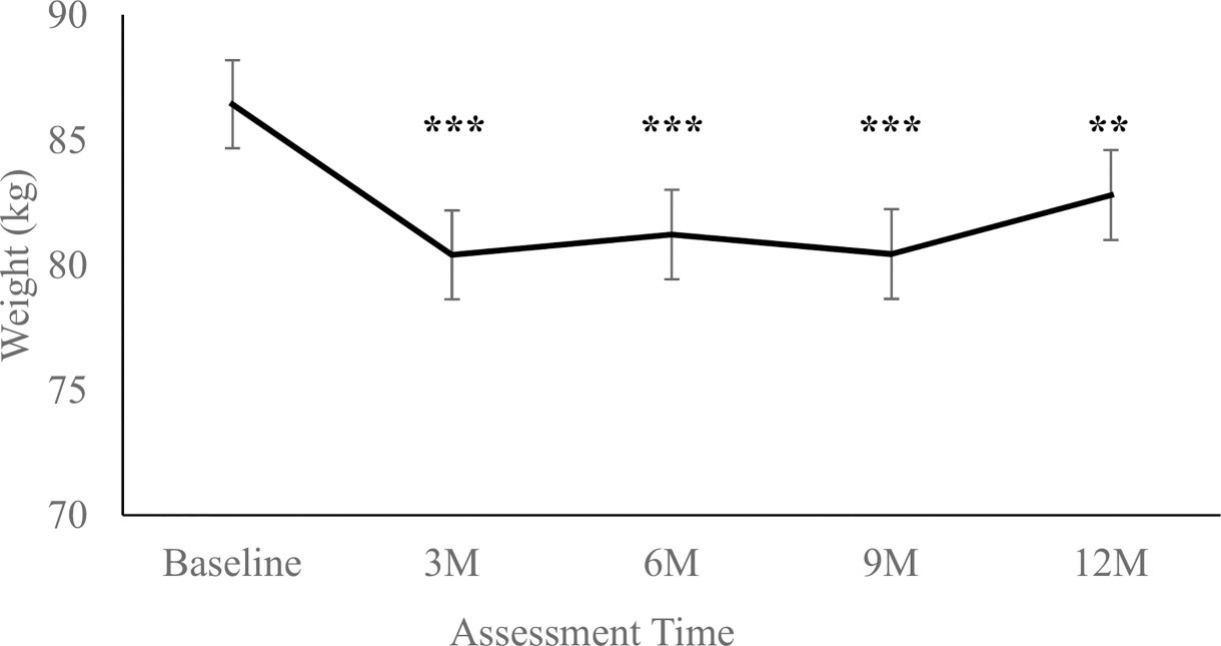
Model-estimated averages (mean ± SE) of participant weight (kg) at each assessment point. Legend: M = Month. *** indicates *p* < .001, ** indicates *p* < .01, and * indicates *p* < .05, compared to baseline.

Retention (defined as attendance at the in-person assessment visit) was 93.3% at Month 3 (n = 70), 90.7% at Month 6 (n = 68), 88.0% at Month 9 (n = 66), and 88.0% at Month 12 (n = 66). Participants lost an average (mean ± SE) of -6.01 ± 1.12 kg during the initial intervention (from baseline to Month 3; see Fig 1), *t*(268) = -5.40, *p* < .0001, representing a model-estimated change from baseline weight of -6.96%. There were not significant changes in weight between Month 3 and Month 6, *p* = .475, or between Month 6 and Month 9, *p* = .501; however, participants experienced significant weight regain between Month 9 and Month 12, *t*(265) = 2.04, *p* = .042. Overall, participants regained an average of 2.39 ± 1.14 kg during the maintenance period (from Month 3 to Month 12), *t*(266) = 2.09, *p* = .037, representing a model-estimated change of 2.97% from Month 3. Overall weight loss from baseline to Month 12 was -3.62 ± 1.14 kg, *t*(268) = -3.18, *p* < .001, representing a model-estimated change of -4.19%.

### Food reward sensitivity

**Fig 2:**
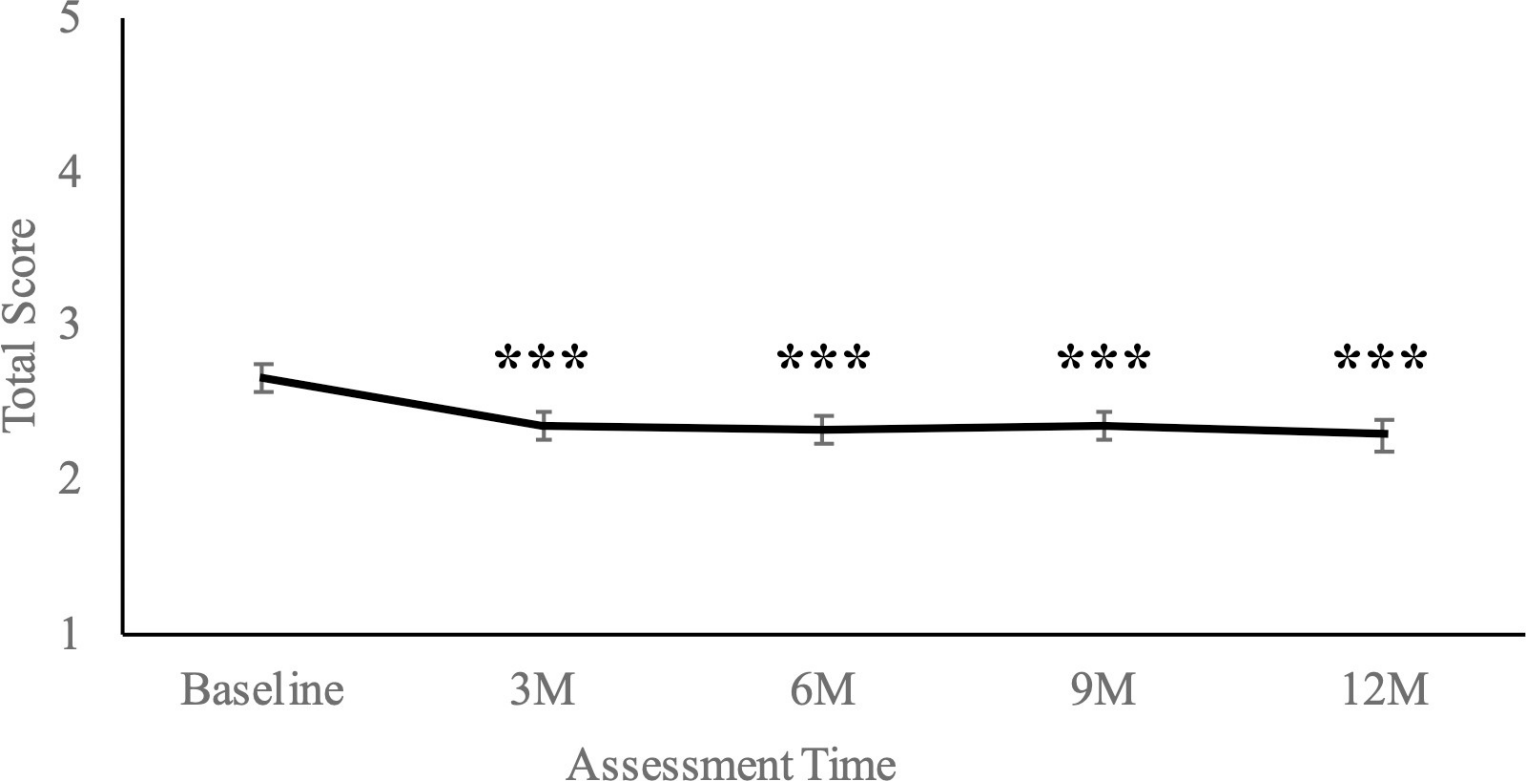
Model-estimated averages (mean ± SE) of total scores on the power of food scale at each assessment point. Legend: M = Month. *** indicates *p* < .001, ** indicates *p* < .01, and * indicates *p* < .05, compared to baseline.

At baseline, the average (mean ± SD) total score on the PFS was 2.66 ± 0.81 points. Fig 2 presents total scores on the PFS at each assessment point. A significant decrease in PFS total score was observed between baseline to Month 3, mean ± SE change = -0.33 ± 0.06 points, *t*(269) = -5.17, *p* < .0001. This decrease was maintained through 12 months, with scores at every later assessment point significantly lower than baseline, all *p*s < .0001. No changes were observed between later time points (i.e., no change in PFS was observed from Month 3 to Month 6, *p* = .820, Month 6 to Month 9, *p* = .674, or Month 9 to Month 12, *p* = .320).

Similar patterns were observed on two of the three PFS subscales. There were significant decreases in Food Available subscale scores from baseline to Month 3 (from a mean ± SE of 2.42 ± 0.20 to 2.04 ± 0.10, respectively, *p* < .0001), but there were no significant changes in scores between assessment visits after Month 3 (Month 3 to 6 *p* = .451, Month 6 to 9 *p* = .521, Month 9 to 12 *p* = .118) and these lower scores were maintained through Month 12 (*p*s < .001 for comparisons between baseline and later time points). There were also significant decreases in scores on the Food Present subscale from baseline to Month 3 (from 3.28 ± 0.11 to 2.67 ± 0.11, respectively, *p* < .0001), but no other changes were observed after Month 3 (Month 3 to 6 *p* = .523, Month 6 to 9 *p* = .771, and Month 9 to 12 *p* = .969); reductions at Month 3 were maintained until Month 12, *p*s < .0001 for comparisons between baseline and later assessment points. A slightly different pattern was observed with scores on the Food Tasted subscale; while an overall decrease in scores on this subscale was observed between baseline and Month 12, from 2.47 ± 0.10 points to 2.33 ± 0.10 points, *p* = .042, there were no significant differences between any intermediate points (baseline to Month 3 *p* = .492, Month 3 to Month 6 *p* = .348, Month 6 to Month 9 *p* = .996, Month 9 to Month 12 *p* = .670).

Across all assessment visits (baseline through Month 12), there was a significant positive association between total PFS scores and weight, *β* = 0.18, *SE* = 0.07, *t*(338) = 2.73, *p* = .007, such that higher scores on the PFS, representing greater food reward sensitivity, were associated with higher weights. There were no significant associations between baseline total PFS scores and weight loss during the intervention period (from baseline to Month 3), *p* = .832, weight regain during the maintenance period (Month 3 to Month 12), *p* = .747, or weight change over the entire study period (from baseline to Month 12), *p* = .595. There were also no significant associations observed between change in total PFS from baseline to Month 3 and initial weight loss during intervention, *p* = .356, weight regain after intervention, *p* = .491, or change in weight over the entire study period, *p* = .114.

### Behavioral impulsivity

**Fig 3:**
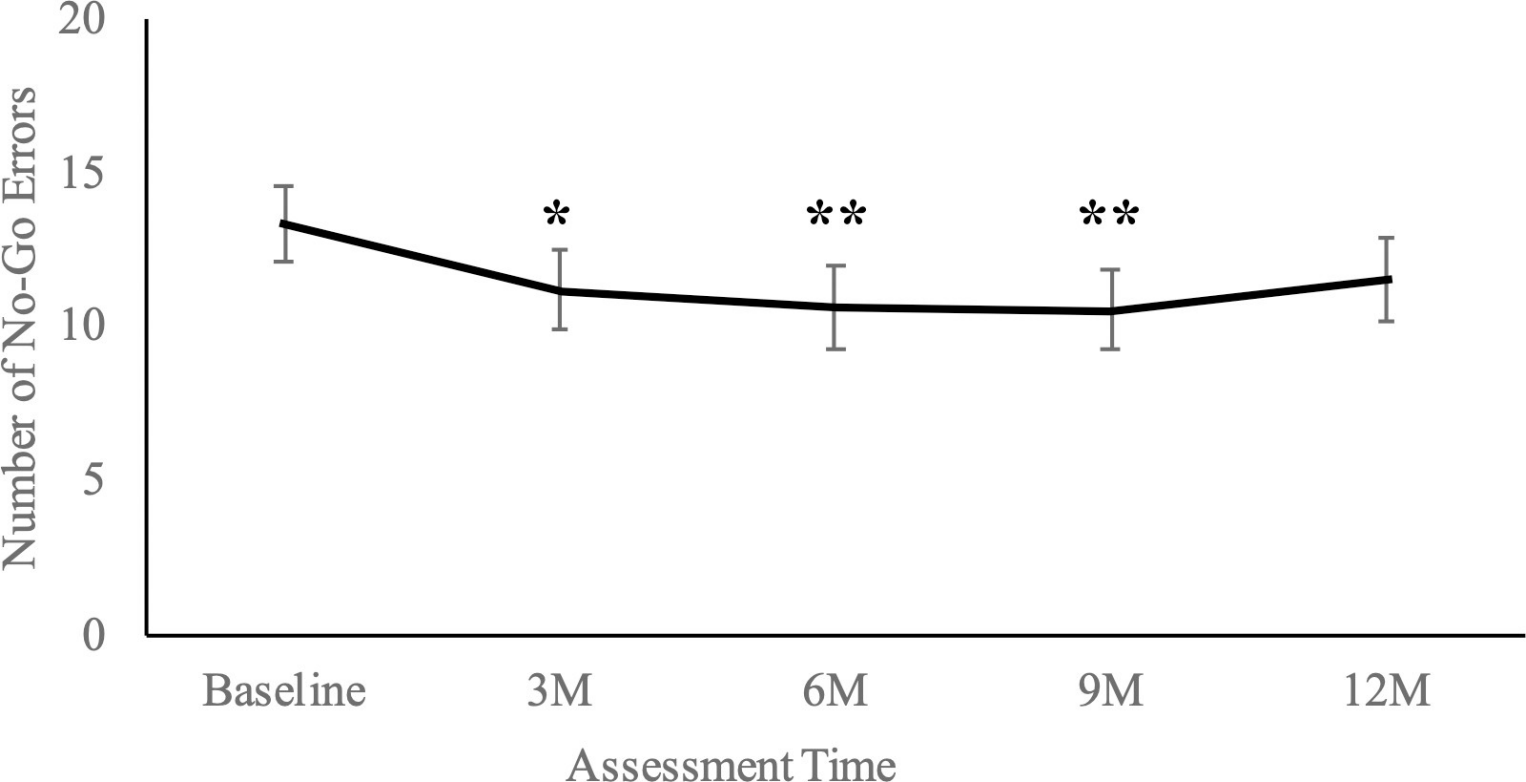
Model-estimated averages (mean ± SE) of total No-Go errors on the Go No-Go task at each assessment point. Legend: M = Month. *** indicates *p* < .001, ** indicates *p* < .01, and * indicates *p* < .05, compared to baseline.

At baseline, participants made an average (mean ± SD) of 13.32 ± 11.39 commission errors on the “No-Go” portion of the Go/No-Go computer task. See Fig 3 for the total number of “No-Go” commission errors made at each time point. There was a significant decrease in No-Go errors from baseline to Month 3, mean ± SE change = -2.16 ± 0.95 errors, *t*(255) = -2.27, *p* = .024, that was maintained until Month 9 (compared to baseline, participants demonstrated fewer errors at both Month 6, *t*(255) = -2.83, *p* = .005, and Month 9, *t*(256) = -2.87, *p* = .005; however, no significant difference was observed between the number of No-Go errors made at baseline and the number of errors made at Month 12, *p* = .088). There were no significant differences between other time points (Month 3 to Month 6 *p* = .570, Month 6 to Month 9 *p* = .934, Month 9 to Month 12 *p* = .350).

Collapsing data across all assessment points, there was not a significant association between number of No-Go errors made and weight, *p* = .116. Moreover, there were no significant associations between No-Go errors made at baseline and weight loss during the initial intervention, *p* = .251, weight regain during the maintenance period, *p* = .700, or total weight change over the course of the study period, *p* = .128. There were also no significant associations observed between change in No-Go errors from baseline to Month 3 and initial weight loss, *p* = .515, weight regain during the maintenance period, *p* = .849, or weight change over the full study year, *p* = .402.

### Delay discounting

**Fig 4:**
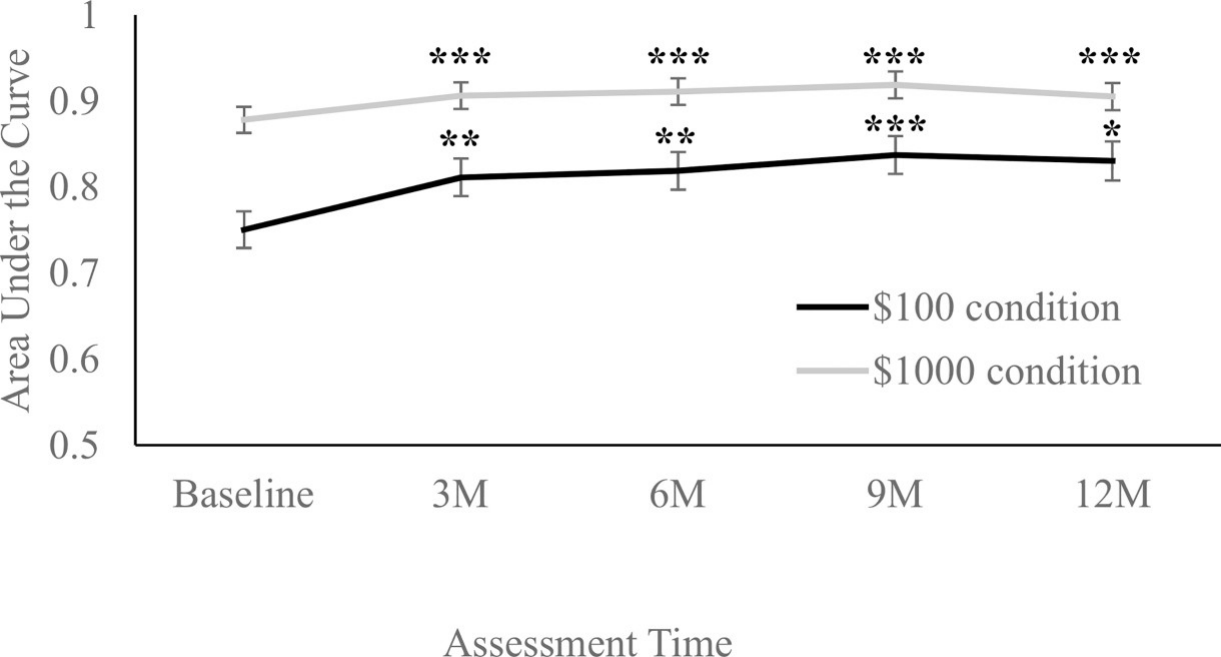
Model-estimated averages (mean ± SE) of delay discounting (calculated via area under the curve) at each assessment point, for both $100 and $1000 conditions. Legend: M = Month. *** indicates *p* < .001, ** indicates *p* < .01, and * indicates *p* < .05, compared to baseline. Higher Areas Under the Curve (AUCs) represent less discounting (and thus less impulsivity).

At baseline, the average (mean ± SD) AUC for delay discounting was 0.75 ± 0.23 for the $100 condition and 0.88 ± 0.15 for the $1000 condition. Fig 4 presents average delay discounting scores at each assessment time point. In the $100 condition, there was a significant improvement in scores (representing less discounting) from baseline to Month 3, mean ± SE change = 0.06 ± 0.02, *t*(252) = 3.78, *p* < .001. While this change was maintained over time (all later time points demonstrated significantly higher AUC values than baseline, all *p*s < .001), there were no other differences observed between intermediate time points (Month 3 to Month 6 *p* = .644, Month 6 to Month 9 *p* = .269, Month 9 to Month 12 *p* = .700). A similar pattern was observed in the $1000 condition; participants experienced a significant improvement in scores from baseline to Month 3, mean ± SE change = 0.03 ± .01, *t*(245) = 2.81, *p* = .005, that was maintained over time (all later time points demonstrated significantly higher AUC values than baseline, all *p*s < .05); however, there were no other differences observed between intermediate time points (Month 3 to 6 *p* = .632, Month 6 to 9 *p* = .446, Month 9 to 12 *p* = .205)

Across all assessment points, there were not significant associations between weight and delay discounting in either the $100 condition, *p* = .655, or the $1000 condition, *p* = .688. There were also no significant associations observed between baseline delay discounting scores in either the $100 or $1000 conditions and initial weight loss, *p*s = .315. and .556, respectively, weight regain during the maintenance period, *p*s = .937 and .705, and overall weight change over the course of the study year, *p*s = .363 and .333. Moreover, there were no significant associations found between change in delay discounting scores in either the $100 or $1000 conditions from baseline to Month 3 and initial weight loss, *p*s = .792 and .913, respectively, weight regain from Month 3 to Month 12, *p*s = .811 and .413, and weight change over the full study year, *p*s = .616 and .477.

### Reinforcement pathology

Table 1 presents correlations between baseline PFS scores, No-Go errors on the Go/No-Go task, and delay discounting scores. While there was a significant correlation between baseline scores on the $100 and $1000 delay discounting conditions, there were not significant correlations between baseline PFS scores, No-Go errors on the Go/No-Go task, and delay discounting scores.

**Table 1:** Correlations between baseline power of food scale scores, No-Go errors on the Go/No-Go task, and delay discounting scores.

Measure	1	2	3
1. Power of Food Scale			
2. No-Go errors on the Go/No-Go	0.05		
3. Delay Discounting, $100 condition	0.23	-0.17	
4. Delay Discounting, $1000 condition	0.20	-0.09	0.64***

*** indicates *p* < .001

** indicates *p* < .01, and

* indicates *p* < .05.

Reinforcement pathology was first defined as the interaction between total PFS score and the number of No-Go errors on the Go/No-Go task. Using this definition, there was not a significant association between reinforcement pathology and weight when collapsing data across all assessment visits, *p* = .889. There were also no significant associations between baseline reinforcement pathology and initial weight loss, *p* = .123, weight regain during the maintenance period, *p* = .174, or total weight change during the full study year, *p* = .251. Moreover, there were no significant associations between the interaction of change in total PFS score and the number of No-Go errors and weight loss from baseline to Month 3, *p* = .993, weight regain from Month 3 to Month 12, *p* = .517, or weight change over the full study year, *p* = .471.

Next, reinforcement pathology was examined as the interaction between total PFS score and delay discounting, using both the $100 and $1000 conditions separately. There was not a significant association observed between reinforcement pathology and weight when collapsing data across all assessment visits, using either the $100 condition, *p* = .749, or the $1000 condition, *p* = .755. There were also no significant associations between these definition of reinforcement pathology and initial weight loss from baseline to Month 3, *p* = .209 for the $100 condition and *p* = .591 for the $100 condition, weight regain from Month 3 to Month 12, *p*s = .306 and .264, respectively, and overall weight change from baseline to Month 12, *p*s = .105 and .133, respectively. Finally, there were no significant associations between the interaction of change in total PFS score and delay discounting, using either the $100 or $1000 conditions, and weight loss from baseline to Month 3, *p*s = .475 and .809 respectively, weight regain from Month 3 to Month 12, *p*s = .509 and .715, or weight change over the full study year, *p*s = .553 and .906.

## Discussion

The current study investigated associations between food reward sensitivity, impulsivity, and weight change during and after a 12-week, Internet-based weight loss program. Participants lost an average of 6 kg during the initial weight loss program and regained about one third of this lost weight during a 9-month observational “maintenance” period. Consistent with hypotheses, higher food reward sensitivity was associated with higher weight when data were collapsed cross-sectionally across time points; in contrast to hypotheses, there were no associations between behavioral impulsivity, delay discounting, or the interactions between food reward sensitivity and either behavioral impulsivity or delay discounting (representing the construct of “reinforcement pathology”) and weight across time points. Moreover, there were no significant associations between baseline values of food reward sensitivity, behavioral impulsivity, delay discounting, or reinforcement pathology and either initial weight loss or weight regain during the maintenance period. Finally, while participants demonstrated decreases in food reward sensitivity, behavioral impulsivity, and delay discounting during the intervention, there were no significant associations between these changes (or changes in reinforcement pathology) and either initial weight loss or later weight regain.

Despite previous findings suggesting that food reward sensitivity, impulsivity, and reinforcement pathology are associated with BMI in cross-sectional studies [2–4,7–10] and can predict palatable food intake in lab-studies [14,15] and weight gain over time in adults without obesity [18], the current results suggest that food reward sensitivity, impulsivity and their interaction may not serve as useful predictors of weight loss outcomes in adults taking part in a weight management program. Similar results were reported in two [19,20] of the three studies [19–21] that have investigated whether baseline food reward sensitivity predicted weight loss in adults; no previous studies have examined whether baseline impulsivity predicts weight loss in adults. This was also the first study to examine whether the interaction between food reward sensitivity and impulsivity predicted weight loss in adults and whether any of these constructs could predict weight regain after the end of initial treatment.

Beyond serving as predictors, our results also suggest that changes in food reward sensitivity, impulsivity, and reinforcement pathology may not be consistent correlates of weight change for individuals taking part in a weight management intervention. While results demonstrated that participants experienced decreases in food reward sensitivity and impulsivity during the weight loss program, consistent with the previous literature [19–21,23,24], these decreases were not associated with weight change. These results are in contrast with two previous studies which found that decreases in food reward sensitivity were associated with greater weight loss in adults [20,21]. Each of these studies, conducted by the same research group and each including 111 participants, demonstrated pre and post-intervention PFS scores similar to those observed in the current study. In each study, however, the magnitude of associations between food reward sensitivity and weight change was small (with *r*s ~ .20). As a secondary analysis of an intervention implementation pilot, the current study may have been underpowered to detect this size of an effect.

Considering alternative interpretations, it could also be possible that food reward sensitivity and impulsivity can be reduced via participation in a weight loss program, but that these changes do not directly influence weight outcomes in adults with overweight and obesity. For example, it is be possible that eating for reasons unrelated to food reward (e.g., emotional eating) may play a larger role in weight regulation for adults with overweight and obesity who choose to enroll in a behavioral weight management program. The observed changes in food reward sensitivity and impulsivity may also have been insufficient to impact weight. Unfortunately, clear guidelines regarding the clinical significance of specific changes in scores on the PFS, the Go/No-Go task, or for delay discounting have not been developed. Future research should establish minimum thresholds for determining meaningful change on these measures.

As another alternative interpretation, participation in a behavioral weight management program may reduce the influence of food reward sensitivity and impulsivity on weight loss outcomes. As a core part of these programs, individuals are taught to self-monitor their food intake in order to meet specific calorie goals [40]; this may shift the focus of eating away from food reward and towards satiety and fullness. Participants are also taught to engage in stimulus control [40], and are encouraged to modify to their food environments in order to support healthy eating habits (e.g., to keep tempting foods out of the house, to plan and prepare meals ahead of time to prevent temptation of higher-calorie fast food or take out options). This skills training component may limit availability of food rewards, thereby reducing potential for impulsive food choices to influence intake. Neither the current study nor any previous studies in this area have included an alternative treatment arm or a no-treatment control condition, precluding investigation into these possibilities. Because a comparison group was not available, the current study and past studies in this field also cannot rule out other processes (such as social desirability, demand characteristics, or testing effects) that could contribute to changes in food reward sensitivity and impulsivity over time. Future studies should investigate changes in these constructs in relation to a control or alternative treatment arm, in order to examine whether decreases in food reward sensitivity and impulsivity observed in this study and other studies are a result of taking part in a weight loss program or an artifact of repeated measurement or demand characteristics.

It may also be possible that differences between our results and the previous literature are driven by differences in methodology and measurement approaches. Previous studies demonstrating cross-sectional associations between impulsivity or reinforcement pathology and obesity have used samples including adults with overweight and obesity in addition to normal weight [7,10,17,23]; our sample included only adults with overweight and obesity, possibly leading to null results due to restriction in range of body weights. Our sample also may not be representative of the larger population of adults with overweight or obesity or of those seeking weight loss treatment. For example, our sample may have had higher motivation for weight loss or lower impulsivity than participants in previous studies. Although the current study did not assess weight loss motivation, the average weight losses observed were smaller than those found in traditional in-person interventions (consistent with the broader tendency for Internet-based interventions to demonstrate smaller weight loss outcomes compared to in-person programs [41]) and similar to those found in previous studies implementing the same Internet-based program in alternative settings [42,43]. Errors of commission on the Go/No-Go task were similar to those observed in other studies including samples of adults with obesity [44,45]. Delay discounting scores in the current study appeared to be higher than those in other studies using a similar task and similar scoring approach (the AUC method) [7,15,16]; however, these other studies used either longer delay periods (of up to 5 years, versus the 1 year used in the current study) and/or smaller amounts of hypothetical rewards (e.g., $10 to $100 instead of the $100 to $1000 used in the current study, and it has been well documented that longer delay periods and smaller rewards lead to greater levels of discounting [10,46,47].

Methods of measuring food reward sensitivity and impulsivity also vary across the literature; while we attempted to use the most commonly-used measures for both food reward sensitivity and impulsivity, it could be that these measures did not adequately capture these constructs. For example, most studies using laboratory tests of food reward sensitivity also assess and control for sensitivity to non-food rewards [2,3,5,14,17]; it could be possible that food reward sensitivity relative to non-food rewards is a more precise measure of this construct than overall food reward sensitivity. It could also be possible that broad impulsivity tasks related to reaction time and monetary discounting may not tap into domains of impulsivity directly related to eating behaviors. Indeed, post-hoc analyses of the current results demonstrated no associations between baseline PFS scores, No-Go errors on the Go/No-Go tasks, or delay discounting, supporting the independence of these constructs. Food-specific tasks might demonstrate a different pattern of outcomes. Recent research has focused on the development of food-based Go/No-Go tasks [48–51], with some studies showing that scores on these tasks correlate with BMI [49,50] and can predict the amount of food consumed in laboratory settings [50]. Moreover, while few studies have investigated delay discounting tasks that involve food instead of money, there is evidence that individuals may discount food (and other consumables) more steeply than money [52–54]. Future studies should investigate whether these tasks may provide better measurement of the constructs of food reward sensitivity and impulsivity as they relate to weight loss.

Finally, differences in study outcomes may also be related to the limited amount of available research in this area. While this could result from the relative novelty of these constructs in the weight management literature (the term “reinforcement pathology” was first used in 2011 [13]), it is also possible that there may be issues in this area related to the “file drawer problem,” such that studies which demonstrated null results were not published [55]. A systematic review of the published and gray literature may prove informative for design of future studies in this area.

Taken together, results suggest that the constructs of food reward sensitivity, impulsivity, and reinforcement pathology may have limited clinical utility within behavioral weight management interventions. Future intervention studies should consider adding in measures of these constructs (especially newer food-based tasks) to examine whether a similar pattern of results is observed in larger, more diverse samples. For novel treatment development, however, greater benefit may be attained by focusing on constructs that have demonstrated robust associations with weight loss and long-term maintenance (e.g., problem solving and self-monitoring skills [29,56,57]).

The current study had several important limitations. First, while we included multiple measures of impulsivity, it is possible that these measures did not appropriately capture impulsivity as it is related to weight management behaviors. It could also be possible that overall executive functioning, rather than impulsivity alone, may be important for predicting weight loss outcomes [58]. Similarly, other measures of food reward sensitivity exist (e.g., laboratory-based tasks that force participants to choose between food versus alternative reinforcers [59]); however, the measure included in the current study has been used previously in this literature and has demonstrated good validity in relation to intake of palatable foods as assessed in laboratory and free-living settings [30]. Second, the parent study was a pilot implementation study and did not include a control group; thus, we cannot establish whether the intervention itself was the cause of beneficial improvements in food reward sensitivity and impulsivity observed between baseline and Month 3. Moreover, power calculations were not conducted to inform recruitment for the parent study and recruitment was limited to a sample of 100 by design [26]; thus the current study may be underpowered to detect effects. Third, the sample of adults included in the current study were predominately White and female, limiting generalizability of findings. Future studies should investigate these constructs within the setting of a randomized controlled trial (in which participants are randomized to either various types of weight loss interventions and/or to a no-treatment control group), investigate potential mechanisms for covariation between food reward sensitivity, impulsivity and weight change, and recruit samples that include more men and adults from racial/ethnic minority groups.

Despite these limitations, the current study had several notable strengths. First, we used multiple measures of impulsivity, which may be particularly important as behavioral impulsivity and delay discounting may have different mechanistic pathways for affecting weight-related behaviors. Second, measures were collected longitudinally, across multiple assessment time points both during and after a weight loss program, allowing us to assess these constructs in relation to both initial weight loss and longer-term weight regain. Finally, the current analyses were strengthened by use of models that could use all available data, overcoming biases introduced by complete-case analyses.

## Conclusion

The current study was the first to investigate the constructs of food reward sensitivity, impulsivity, and reinforcement pathology in the context of a behavioral weight loss program. Results demonstrated that, across time points, greater food reward sensitivity was significantly associated with higher body weight; however, there were no significant associations across time between either behavioral impulsivity or delay discounting and weight, and no evidence supporting an interaction between food reward sensitivity and either measure of impulsivity (representing the construct of “reinforcement pathology”). Significant decreases in food reward sensitivity, behavioral impulsivity, and delay discounting were observed during the intervention period (baseline to Month 3); however, neither baseline values of these constructs nor these changes over time were associated with either initial weight change during the intervention or weight regain during a post-intervention maintenance period (Month 3 to Month 12). Similarly, there were no associations between baseline reinforcement pathology and either initial weight loss or later weight regain. While these results were unexpected and contrary to hypotheses, much of the literature used to support initial hypotheses included studies that were either cross-sectional, laboratory-based, or had small samples and did not adequately control for missing data. Taken together, results suggest that the broad constructs of food reward sensitivity, impulsivity, and reinforcement pathology may have limited clinical utility within behavioral weight management interventions. Future intervention studies should consider adding food-related measures of these constructs (e.g., tasks that measure impulsivity specifically to food cues and food-related delay discounting) to examine whether a similar pattern of results is observed.

## Supporting information

S1 Data Full dataset used for all study analyses. (XLSX)
